# A novel role for the DNA repair gene *Rad51* in Netrin-1 signalling

**DOI:** 10.1038/srep39823

**Published:** 2017-01-06

**Authors:** K. A. Glendining, D. Markie, R. J. M. Gardner, E. A. Franz, S. P. Robertson, C. L. Jasoni

**Affiliations:** 1 Brain Health Research Centre, Department of Anatomy, University of Otago, Dunedin, New Zealand; 2 Department of Pathology, University of Otago, Dunedin, New Zealand; 3 Clinical Genetics Group, Department of Women’s and Children’s Health, University of Otago, Dunedin, New Zealand; 4 Department of Psychology and fMRIOtago, University of Otago, Dunedin, New Zealand

## Abstract

Mutations in *RAD51* have recently been linked to human Congenital Mirror Movements (CMM), a developmental disorder of the motor system. The only gene previously linked to CMM encodes the Netrin-1 receptor DCC, which is important for formation of corticospinal and callosal axon tracts. Thus, we hypothesised that Rad51 has a novel role in Netrin-1-mediated axon development. In mouse primary motor cortex neurons, Rad51 protein was redistributed distally down the axon in response to Netrin-1, further suggesting a functional link between the two. We next manipulated Rad51 expression, and assessed Netrin-1 responsiveness. *Rad51* siRNA knockdown exaggerated Netrin-1-mediated neurite branching and filopodia formation. RAD51 overexpression inhibited these responses, whereas overexpression of the CMM-linked R250Q mutation, a predicted loss-of-function, had no effect. Thus, Rad51 appears to negatively regulate Netrin-1 signalling. Finally, we examined whether Rad51 might operate by modulating the expression of the Unc5 family, known negative regulators of Netrin-1-responsiveness. *Unc5b* and *Unc5c* transcripts were downregulated in response to Rad51 knockdown, and upregulated with RAD51 overexpression, but not R250Q. Thus, Rad51 negatively regulates Netrin-1 signalling, at least in part, by modulating the expression of Unc5s. Imbalance of positive and negative influences is likely to lead to aberrant motor system development resulting in CMMs.

The DNA repair gene *RAD51* has been previously reported to maintain genetic stability during cell division through homologous recombination, by performing repair of double strand DNA breaks, and regulating cell cycle checkpoint mechanisms to prevent the replication of defective DNA and accumulation of mutations^1^,^2^. In vertebrate cells, *Rad51* is essential for proliferation; cells deficient in *Rad51* amass chromosomal abnormalities before eventual cell death^1^, and genetic deletion is embryonic lethal in mice^3^,^4^. Considering these known functions for maintaining DNA integrity, it was unanticipated when recent studies implicated mutations in *RAD51* with both sporadic and familial cases of congenital mirror movements (CMM)^5^,^6^,^7^.

CMM are involuntary mirror reversals of movements occurring on one side of the body when voluntary movements on the opposite side of the body are performed, typically occurring in the hands^8^,^9^. Anomalous projections of motor systems are implicated in this disorder, for example, studies using focal transcranial magnetic stimulation (TMS) have revealed that in people with CMM, unilateral motor cortex stimulation evokes bilateral motor potentials in the muscles of the hands, suggesting abnormal ipsilateral corticospinal tract (CST) projections from motor cortex hand areas to the spinal cord (for review see^10^). In addition, recording of scalp potentials during movement preparation, and interference with the cortical motor output by focal TMS, have shown bilateral activation of the motor cortex during intended movements, indicating abnormal callosal inter-hemispheric inhibition^10^,^11^. These findings indicate that CMM are likely underpinned by abnormal development of the decussating CST and/or corpus callosum^11^,^12^,^13^. In support of this, genetic analysis of people with CMM have identified mutations in *Deleted in Colorectal Cancer* (*DCC*)^5^,^14^,^15^, a receptor for Netrin-1, which is an axon guidance molecule known to be important for midline guidance decisions during development, including decussation of the CST and CC^16^,^17^ as well as neuronal branching and filopodia formation of the axons that form these tracts^18^.

*Dcc* knockout mice display callosal agenesis^19^, and conditional *Dcc* mutants display a hopping gait, where the hind limbs are unable to move independently^20^, a motor phenotype that has been likened to mirror movements. Thus, whereas a deficit in DCC function correlates well with the features of CMM, the association with RAD51 is intriguingly less clear. The role of Rad51 in DNA repair in S and G_2_ phases of division is well documented^21^,^22^,^23^,^24^, however there is no described function in post-mitotic, G_0_ phase, terminally differentiated neurons, nor a role in motor system development. Although Rad51 can be detected in the brain throughout development, and is expressed within neural progenitor cells in the developing mouse neocortex from as early as E12 through to post natal stages^6^, it is unknown whether Rad51 serves any additional cellular function outside of DNA repair.

In light of the association of Rad51 mutation with CMM, we hypothesised that Rad51 may play a novel, as yet undescribed role in Netrin-1 signalling in the development of post-mitotic neurons within the motor system.

## Results

### Rad51 was redistributed within neuronal cytoplasm in response to Netrin-1

**Figure 1:**
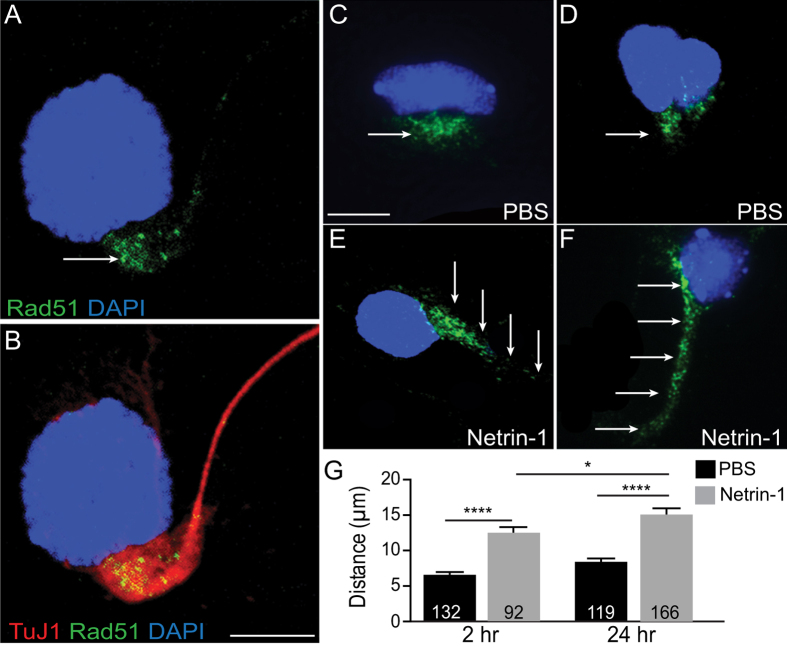
Rad51 was redistributed in cortical neurons in response to Netrin-1. (**A**) Representative confocal image of a cultured E14.5 mouse motor cortical neuron immunolabelled for Rad51 (green) and (**B**) merged with immunolabel for pan neuronal marker TuJ1 (red). Nuclei are counterstained with DAPI (blue). Arrow indicates cytoplasmic Rad51 immunolocalisation aggregated in a peri-nuclear pattern of expression. Scale bar is 5 μm. (**C–F**) Confocal images of E14.5 mouse motor cortical neurons following treatment with PBS vehicle or Netrin-1 for 2hr (**C,E**) or 24 hr (**D,F**). Nuclei are counterstained with DAPI (blue). Arrows depict immunolocalisation of Rad51. Scale bar is 5 μm. (**G**) Bar graph showing quantification of Rad51 axonal redistribution measured as distance from the nucleus (μm) following 2 or 24 hour treatment with vehicle (PBS) or Netrin-1. Values are mean ± SEM, *p < 0.05, **p < 0.001, ****p < 0.00001.

Genetic screening of people with CMM has identified mutations in *RAD51*^6^,^7^, or missense/truncating mutations in *DCC*, a Netrin-1 receptor^14^,^15^. The shared CMM phenotype from mutations affecting either of these genes lead us to hypothesize that RAD51 may be involved in Netrin-1 signalling. To investigate this, Rad51 subcellular distribution and expression levels were examined in primary neuron cultures from E14.5 mouse motor cortex using a Rad51 antibody, and co-labelling with neuron-specific beta-III tubulin (TuJ1). Rad51 immunoreactivity showed strong perinuclear aggregation in all TuJ1-positive, post-mitotic neurons (Fig. 1A,B). This contrasts with previous reports that describe a diffuse distribution of Rad51 through the cytoplasm and/or nucleus of neuronal, post-mitotic cells^6^,^25^.

Next, we examined neuronal Rad51 localisation following the application of recombinant mouse Netrin-1 (200 ng/mL) or vehicle (PBS). Netrin-1 treatment stimulated a redistribution of Rad51 protein within the neuronal cytoplasm, away from the border of the nucleus and extending distally down the primary axon at both 2 hr and 24 hr post-Netrin-1 treatment (Fig. 1C–F). This change was absent in PBS-treated neurons, which retained a perinuclear pattern of Rad51 expression (Fig. 1C,D). Significant redistribution of Rad51 within the neuronal cytoplasm could be observed after 2 hr Netrin-1 treatment, with Rad51 immunolabel detected nearly twice the distance from the nucleus compared to PBS-treated neurons (Fig. 1C,E,G; Netrin-1: 12.48 μm ± 0.838, n = 92; PBS: 6.569 μm ± 0.412, n = 132; Unpaired t test with Welch’s correction p < 0.0001).

Twenty-four hours after Netrin-1 treatment, significant Rad51 redistribution away from the nucleus persisted (Fig. 1D,F,G; Netrin-1: 15.11 μm ± 0.860, n = 166; PBS: 8.414 μm ± 0.473, n = 119, p < 0.0001; Unpaired t test with Welch’s correction), extending further down the axon compared with 2 hr post-Netrin-1 (Fig. 1E,F,G, p < 0.05).

Quantification with qPCR showed no difference in *Rad51* mRNA expression between Netrin-1- and PBS-treated cortical cultures at 2 hr or 24 hr, and no change to Rad51 protein abundance was detected by Western blotting after 24 hr Netrin-1 treatment (see Supplementary Fig. S1), indicating that a redistribution of existing cytoplasmic stores of Rad51 occurs in response to Netrin-1, as opposed to stimulation of transcription or translation. Together, these observations suggest a novel, non-canonical function for Rad51 in post-mitotic cortical neurons associated with Netrin-1 signalling, and distinct from its role in facilitating DNA damage repair in mitotic cells.

### Knockdown of Rad51 in primary cortical neurons exaggerated Netrin-1-induced branching

Previous studies have shown that application of Netrin-1 to cortical neuron cultures results in increased filopodia formation and axonal branching^26^,^27^. To determine whether Rad51 might be involved in these Netrin-1-induced changes we performed siRNA-mediated knockdown of *Rad51*, and examined axon length, neurite number, and filopodia formation in both the presence and absence of Netrin-1 for 24 hr.

To confirm knockdown of *Rad51*, we first screened the efficacy of several siRNAs targeted to either the 5′ UTR or coding region of *Rad51*, and validated their ability to reduce the levels of *Rad51* mRNA and protein in cultured cortical neurons (see Supplementary Fig. S2). This analysis also allowed us to further validate the specificity of the Rad51 antibody. After selection of the most effective *Rad51* siRNA, E14.5 cortical neurons were treated with either siRNA specific for *Rad51* (*Rad51* siRNA), a scrambled control siRNA (scram siRNA), or Lipofectamine transfection reagent alone (vehicle). After 2 DIV, which was sufficient time for significant siRNA-mediated reduction of *Rad51* mRNA and protein (see Supplementary Fig. S2), cortical cultures were exposed to either Netrin-1 or PBS for 24 hr, and axon length, branching, and filopodial formation were assayed.

**Figure 2:**
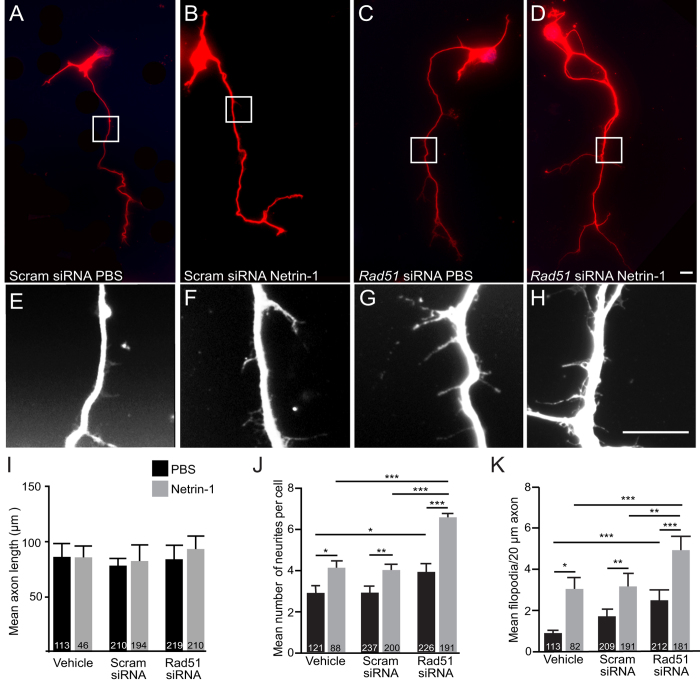
Knockdown of Rad51 with siRNA stimulated Netrin-1 induced axonal branching. (**A–D**) Representative images of E14.5 cortical neurons transduced with siRNA oligonucleotides targeting Rad51 (Rad51 siRNA) or scramble control (Scram siRNA) and labelled with TuJ1 antibody. (**E–H**) Higher magnification images of boxed areas in (**A–D**). Scale bars are 10 μm. (**I–K**) Bar graphs showing comparison of branching dynamics in Vehicle (PBS), scrambled control siRNA, or *Rad51*siRNA-treated neurons following application of PBS or Netrin-1 in relation to (**I**) axon length (μm), (**J**) neurite number, or (**K**) filopodia per 20 μm of axon. Values are mean ± SEM, numbers of neurons analysed are depicted at base of corresponding bars, *p < 0.05, **p < 0.001, ***p < 0.0001.

Consistent with other *in vitro* studies^12^,^18^,^26^, axon length was not altered in response to Netrin-1 in any treatment group, nor was there a difference in total primary axon length between *Rad51*siRNA-treated neurons and scramsiRNA or vehicle controls (Fig. 2A–D and I) at baseline (i.e., without Netrin-1) or in the presence of Netrin-1. Cell-staging analysis verified that the ability of cells to form an axon did not differ across treatment groups (see Supplementary Fig. S3). Thus, depletion of *Rad51* mRNA and protein does not interfere with axonal outgrowth.

In the scram siRNA and vehicle control groups, 24 hr Netrin-1 treatment increased the number of neurites in comparison to PBS-treated control cells (Fig. 2A,C,J; vehicle/Netrin-1: 4.14 ± 0.30, n = 88 versus vehicle/PBS: 2.95 ± 0.15, n = 121, p < 0.001; scram siRNA/Netrin-1: 4.059 ± 0.21, n = 200 versus scram siRNA/PBS: 2.90 ± 0.15, n = 237, p < 0.001), and increased filopodia formation (Fig. 2E,G,K; vehicle/Netrin-1: 3.07 ± 0.24, n = 82 versus vehicle/PBS: 1.43 ± 0.10, n = 113, p < 0.001; scram siRNA/Netrin-1: 3.065 ± 0.17, n = 191 versus scram siRNA/PBS: 1.79 ± 0.08, n = 237, p < 0.001). Strikingly however, following knockdown of *Rad51* the Netrin-1-induced branching response of cortical neurons was exaggerated (Fig. 2B,D,J) (*Rad51* siRNA/Netrin-1: 6.51 ± 0.37, n = 191 versus *Rad51* siRNA/PBS: 3.90 ± 0.22, n = 226, p < 0.001) with Netrin-1-induced neurite branching significantly increased by treatment with *Rad51* siRNA in comparison to both scram siRNA/Netrin-1 (p < 0.001) and Vehicle/Netrin-1 (p < 0.001). Similarly, filopodia formation in response to Netrin-1 was also significantly increased in cultures treated with *Rad51* siRNA compared to scram siRNA (p < 0.001), and vehicle (p < 0.001) (Fig. 2F,H,K). Moreover, in the absence of Netrin-1, *Rad51*siRNA-treated neurons exhibited a greater number of neurites (p < 0.05) and filopodia (vehicle < 0.001) than either scram siRNA or vehicle alone. These data suggest that Rad51 may be a negative regulator of Netrin-1 signalling in the modulation of axonal branching and filopodia formation.

### Overexpression of wild-type RAD51, but not R250Q RAD51, diminished neurite branching of cortical neurons

**Figure 3:**
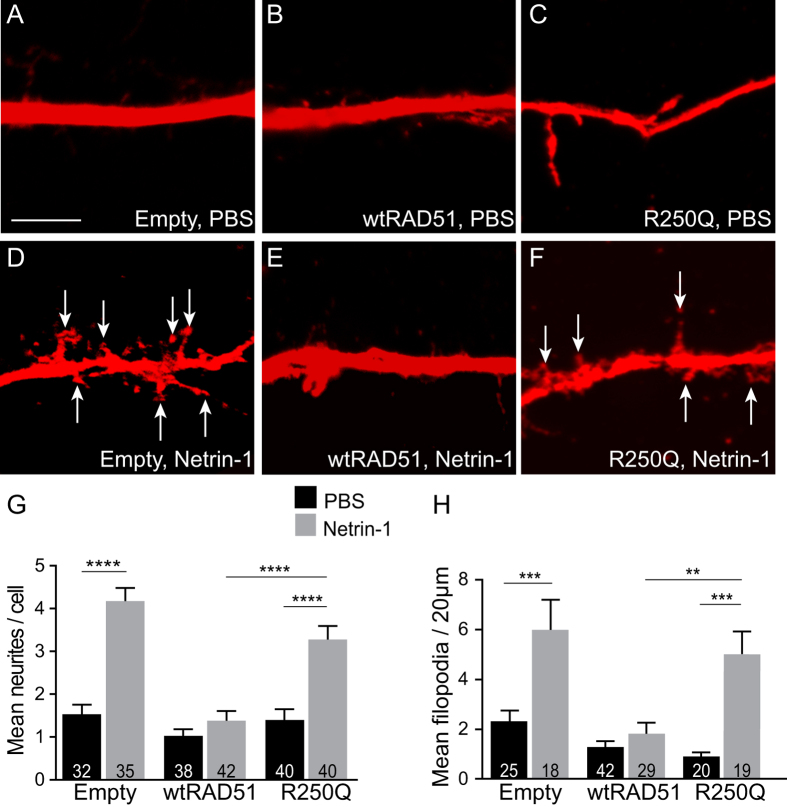
Overexpression of Rad51 abolished axonal branching in response to Netrin-1. Representative images of TuJ1-immunostained axons (red) treated with PBS (**A–C**) or Netrin-1 (**D–F**) following transfection with EYFP fused to an empty plasmid (Empty, **A,D**), wildtype RAD51 (wtRAD51, **B,E**), or the RAD51 R250Q mutant (R250Q, **C,F**). Scale bar is 5 μm. Note that images with TuJ1 and EYFP co-staining are presented in Supplementary Fig. S4. (**G**) Bar graph showing quantification of Netrin-1 induced neuritogenesis from neurons expressing the different constructs, and (**H**) as in (**G**) but showing quantification of filopodia number per 20 μm of axon. Values are mean ± SEM, *p < 0.05, **p < 0.001, ***p < 0.0001.

If Rad51 is indeed a negative regulator of neurite branching and filopodia formation, we predicted that overexpression of RAD51 would inhibit these processes either independently, in response to Netrin-1, or both. In addition, we hypothesised that the RAD51-R250Q mutation associated with CMM may impede this function. To examine this, E14.5 cortical neurons were cultured as previously, and Netrin-1-induced branching response was assessed following transfection with either an empty expression vector encoding only EYFP (Empty, control), an EYFP-wild-type RAD51 fusion construct (wtRAD51), or an EYFP fusion construct containing the naturally occurring human R250Q RAD51 mutation (R250Q). In neurons transfected with EYFP alone, Netrin-1 induced a significant increase of neurites per cell in comparison to PBS treatment (Fig. 3A,D,G; Empty/PBS: 1.531 ± 0.224, n = 32 versus Empty/Netrin-1: 4.171 ± 0.311, n = 35, p < 0.0001). By contrast, neurons expressing wtRAD51 and treated with Netrin-1 had significantly fewer branches than Netrin-1 treated empty vector controls (p < 0.0001), and did not differ significantly from wtRAD51 PBS control (Fig. 3B,E; wtRAD51/PBS: 1.026 ± 0.153, n = 38 versus wtRAD51/Netrin-1: 1.381 ± 0.226, n = 42, p > 0.05). In addition to impaired neuritogenesis, overexpression of wtRAD51 reduced the ability of neurons to form filopodia in response to Netrin-1. Whereas Netrin-1 significantly increased filopodia in neurons transfected with EYFP alone (Fig. 3A,B,H; Empty/PBS: 2.2 ± 0.418, n = 25 versus Empty/Netrin-1: 5.702 ± 1.156, n = 18, p < 0.001), Netrin-1 stimulation of wtRAD51-expressing neurons induced no difference in the number of filopodia per 20 μm of axon length compared with PBS-treated neurons (Fig. 3B,E,H; wtRAD51/PBS: 1.213 ± 0.233, n = 42 versus wtRAD51/Netrin-1: 1.733 ± 0.415, n = 29, p > 0.05). wtRAD51 neurons treated with Netrin-1 also had significantly fewer filopodia in comparison to Netrin-1-treated EYFP controls (Fig. 3D,E,H; Empty/Netrin-1: 5.702 ± 1.156, n = 18 versus wtRAD51/Netrin-1: 1.733 ± 0.415, n = 29, p < 0.0001). By contrast, overexpression of the naturally occurring RAD51 mutant R250Q, a predicted loss-of-function, did not inhibit the Netrin-1-induced branching response. Cortical neurons expressing R250Q and treated with Netrin-1 showed increases in neurite number (Fig. 3C,F,G; R250Q/PBS: 1.400 ± 0.248, n = 40 versus R250Q/Netrin-1: 3.275 ± 0.318, n = 40, p < 0.0001), and significantly more filopodia (Fig. 3C,F,H; R250Q/PBS: 0.857 ± 0.160, n = 20 versus R250Q/Netrin-1: 4.775 ± 0.867, n = 19, p < 0. 001), which did not differ from the branching of EYP-empty vector control neurons treated with Netrin-1 (p > 0.05). These observations indicate that Rad51 functions normally as a negative regulator of Netrin-1-induced filopodia formation and neurite branching in motor cortical neurons. Furthermore, these data suggest that the naturally occurring human mutant RAD51R250Q behaves as a loss-of-function mutant that has lost the ability to oppose this Netrin-1-induced neurite-promoting response in cortical neurons.

### Overexpression or knockdown of Rad51 in cortical neurons altered the expression of Netrin-1 receptors

**Figure 4:**
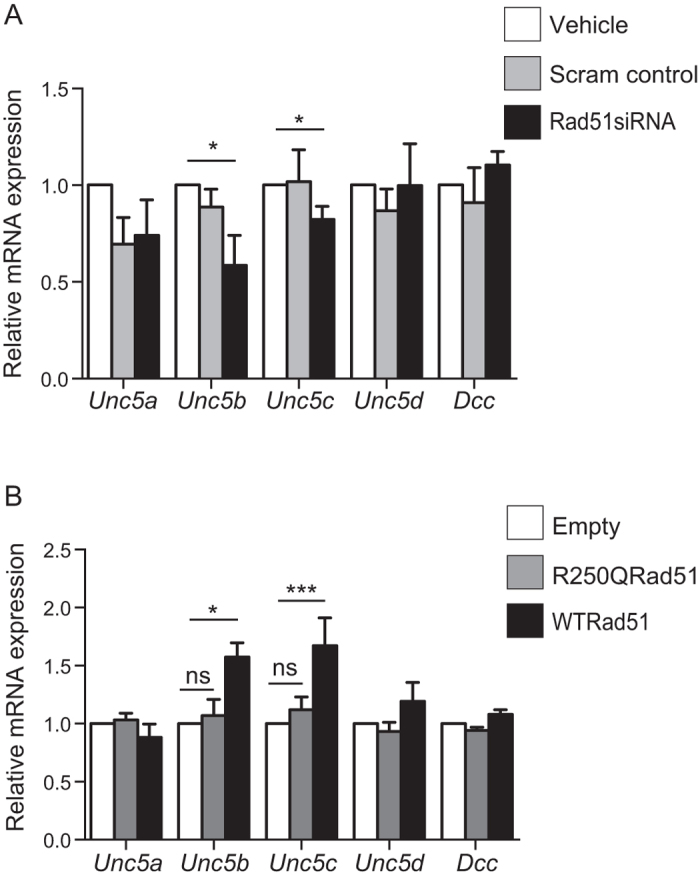
Manipulation of Rad51 expression levels altered gene expression of Netrin-1 receptors. (**A**) Bar graph showing qPCR quantification of Netrin-1 receptor mRNA expression in neurons targeted with *Rad51*siRNA, Scram siRNA (control) or vehicle. (**B**) Bar graph showing qPCR quantification of Netrin-1 receptor mRNA expression in neurons transfected with EYFP, EYFP-RAD51, or EYFP-R250Q. Values shown are mean ± SEM. *p < 0.05.

Given that manipulation of Rad51 expression impacted the branching of neurons in response to Netrin-1 in a manner that suggests it plays a role in opposing the stimulatory effects of Netrin-1 on neuritogenesis, we examined whether this might be due to alterations in the expression of Netrin-1 receptors. Whereas Dcc typically has been reported to mediate the stimulatory effects of Netrin-1 on neuritogenesis. the Unc5 family has been reported to oppose this action^26^,^28^,^29^. Using qPCR we measured relative mRNA levels of *Unc5a-d* and *Dcc* in cultured neurons following siRNA mediated knockdown of *Rad51* (Fig. 4A). No significant differences were observed in *Unc5a-d* or *Dcc* mRNA expression levels between the vehicle or scram siRNA-treated cells (p > 0.05), however the *Rad51* siRNA-treated neurons showed a significant downregulation of both *Unc5b* and *Unc5c* transcript levels in comparison to control (Fig. 4B; *Unc5b*; 0.59 ± 0.155 fold change, n = 4, p < 0.05, *Unc5c*; 0.8236 ± 0.06 fold change, n = 4, p < 0.05), with no change in *Unc5a* (0.74 ± 0.18 fold change, n = 3, p > 0.05), *Unc5d* (0.99 ± 0.22 fold change, n = 4, p > 0.05), or *Dcc* mRNA (1.11 ± 0.069 fold change, n = 5, p > 0.05).

We also quantified relative mRNA levels of *Unc5a-d* and *Dcc* in neurons transfected with EYFP alone, wtRAD51, or R250Q (Fig. 4B). In comparison to neurons expressing EYFP alone, overexpression of wtRAD51 increased the expression of *Unc5b* and *Unc5c* mRNAs (Fig. 4B; *Unc5b*;1.57 ± 0.123 fold change, n = 8, p < 0.0001, *Unc5c*; 1.67 ± 0.24 fold change, n = 7, p < 0.05). There was no change in *Unc5a* (0.88 ± 0.114 fold change, n = 8, p > 0.05), *Unc5d* (1.19 ± 0.163 fold change, n = 10, p > 0.05), or *Dcc* mRNA (1.08 ± 0.040 fold change, n = 6, p > 0.05) in comparison to control. Interestingly, the relative mRNA expression of *Unc5a-d* and *Dcc* in neurons transfected with the R250Q construct did not differ from EYP control (*Unc5a* 1.031 ± 0.05990 n = 5, *Unc5b* 1.070 ± 0.1386 n = 5 *Unc5c* 1.120 ± 0.1113 n = 5 *Unc5d* 0.9328 ± 0.07761 n = 5, p > 0.05), or *Dcc* (0.9413 ± 0.02671 n = 5, p > 0.05), indicating that any regulatory function of Rad51 on Unc5 receptor expression may be lost as a result of the R250Q mutation.

## Discussion

The DNA repair gene *RAD51* acts to maintain genetic stability and prevent the replication of defective DNA in dividing cells. Curiously, mutations in *RAD51* have recently been linked to CMM^5^,^6^,^7^, a developmental disorder of the motor system previously only attributed to deficits in Netrin-1/DCC signalling. Based on this we hypothesized that Rad51 is functionally involved in Netrin-1’s effects on motor system development. We tested this hypothesis using a primary culture model of embryonic mouse neurons from motor cortex, which we challenged with Netrin-1 under conditions in which Rad51 expression and functionality were modulated. Our results indicate that Rad51 serves a novel, non-canonical function in neurons of the motor system, and that this role is in part linked to Netrin-1 regulation of axon branching.

Although Rad51 expression can be detected in neuronal and non-neuronal cells in the brain, its cellular distribution has been described as weak or diffuse throughout the nucleus and/or cytoplasm^6^,^30^. In contrast, we observed Rad51 residing cytoplasmically in a perinuclear location within cortical neurons *in vitro*. In the developing brain, Rad51 is very strongly expressed in the nuclei of proliferative cells^6^, thus detection of lower levels of cytoplasmic Rad51 may have been difficult in tissue. In addition, we observed that Netrin-1 triggered Rad51 to redistribute within the cytoplasm from a perinuclear aggregation to a more dispersed distribution extending distally down the axon, with no concomitant changes in neuronal levels of Rad51 protein or mRNA. These observations suggest that in cortical neurons, a reorganization of existing stores of Rad51 protein occurs in response to Netrin-1. This suggests a functional link between Rad51 and Netrin-1, and further supports the hypothesis.

In light of the reported CST and/or CC tract malformations in CMM patients^11^, and Rad51 responsiveness to Netrin-1, we examined further whether Rad51 is involved in axonal branching, filopodia growth, or axon morphology of neurons from motor cortex, and whether such activities might be functionally linked with Netrin-1-mediated axon development. Interestingly, siRNA knock-down of *Rad51* in the absence of Netrin-1 resulted in increased branching and filopodia formation to approximately the same extent as Netrin-1 in controls. When *Rad51* was knocked-down in the presence of Netrin-1 however, a synergistic response was observed, where neurons with reduced expression of *Rad51* displayed exaggerated branching in response to Netrin-1. Conversely, overexpression of RAD51 was found to have the opposite effect to knockdown, with a decrease in Netrin-1-induced branching. Neurons overexpressing Rad51 did not differ in the baseline number of neurites or filopodia when compared to controls, however in the absence of Netrin-1, neurons exhibited relatively few neurites and filopodia, so any further decrease may be biologically negligible and/or technically difficult to detect. These data suggest that Rad51 may act as a negative regulator of Netrin-1 signalling to modulate axonal branching and filopodia formation of cortical neurons in the motor cortex. Additionally, RAD51 containing the R250Q mutation failed to inhibit Netrin-1 induced branching, supporting bioinformatics predictions that the mutation results in a loss of function. A loss of negative regulation of Netrin-1 signalling would be predicted to cause increased axonal branching of neurons, following exposure to Netrin-1 *in vivo*, leading to anatomical aberrations in the motor system. Functionally, this could result in reduced interhemispheric inhibition between cortical motor areas, lead to the persistence of both contralateral- and ipsilateral-projecting CST branches, or otherwise perturb the balance between side-specific activation and inhibition of a motor programme, resulting in the unwanted movements characteristic of CMM. Neurons transfected with the R250Q construct did not show an increase in neurite or filopodia formation above EYFP-controls, which suggests that the mutation specifically affects Rad51’s Netrin-1-responsiveness, rather than a generic function in axon dynamics. Furthermore, the R250Q mutation is associated with CMM patients who present with no comorbidities, so the data suggests that the loss of Rad51 function associated with the R250Q mutation pertains specifically to its role in Netrin-1 signaling, and not its DNA repair functions.

In an attempt to ascertain a mechanism by which Rad51 might be having its effects on Netrin-1 signaling, we examined whether it might regulate the expression of genes encoding known stimulatory and inhibitory mediators of the Netrin-1 effects on axon dynamics. To this end, we have shown that neurons under- or over-expressing Rad51 exhibit changes in their mRNA expression profile of the Unc5 family of receptors, indicating a potential mechanism for Rad51 modulation of Netrin-1-induced branching. Specifically, Rad51 knockdown downregulated *Unc5b* and *Unc5c* receptor mRNA, whereas overexpression of wildtype RAD51 upregulated these receptor mRNAs. The increase in *Unc5b* and *Unc5c* transcript was absent in neurons overexpressing the R250Q construct, consistent with this mutant lacking Netrin-1 responsiveness. Owing to cellular heterogeneity (only a subpopulation of cortical neurons at this age would be Netrin-1-responsive), and transfection efficiency less than 100%, we believe that the somewhat modest expression changes we observe *in vitro* are likely to be an underestimate of what happens in a single Netrin-1-responsive cell *in vivo*.

Members of the Unc5 family of receptors are repulsive receptors for Netrin-1, negatively regulating filopodia dynamics and mediating growth cone collapse, with knockdown of either Unc5b or Unc5c associated with axon misguidance phenotypes^31^,^32^,^33^. In the developing cortical plate, *Unc5c* is expressed in a high-lateral to low-medial gradient to facilitate guidance of the interhemispheric projection^34^. In addition, Netrin-1 signalling via the Un5c receptors is important for the correct targeting of the CST, at the pyramidal decussation where CST axons traverse contralaterally in the caudal medulla, and at the dorsal funiculus in the spinal cord^20^. Thus, if *Rad51* haploinsufficiency in CMM is similarly associated with disruption to Unc5 expression, then incorrect guidance of the CC and/or CST axons across the midline during development is likely to result. While there is no information available on Unc5 receptor mRNA levels in people with CMM affected by a *Rad51* mutation, this would be a valuable area for further study.

Initially it may seem counterintuitive that mutations in either DCC or RAD51 can result in a CMM phenotype, given that they appear to work in an opposing manner on Netrin-1 signaling^35^. Formation of the CC is reliant on both attractive and repulsive signals however, and recent work suggests that *Unc5c* and *Dcc* might be required at different developmental timepoints for the correct guidance of callosal axons. For example, early pioneer axons from the cingulate cortex express *Dcc* and are attracted by Netrin-1 at the midline to cross into the contralateral hemisphere, whereas later born neurons from Layer V of the cortex express *Unc5c* and are repelled away from Netrin-1 at the internal capsule^34^, thus both attractive and repulsive signalling contribute to the midline crossing of callosal axons.

In conclusion, we have identified a novel role for Rad51, previously known only for its role in DNA repair. Our data suggest that Rad51 acts as a negative regulator of Netrin-1 signalling during development; a function that is consistent with observations in humans that *RAD51* haploinsufficiency can lead to developmental defects in control of bilateral movements. These results add another player to the collection of positive and negative factors that work in concert to orchestrate proper neural development.

## Methods

### Cell Culture

All procedures described in this study were approved by the University of Otago, Animal Ethics Committee in accordance with the guidelines on Care and Use of Laboratory Animals (NIH Publication No. 85–23, 1996), and in accordance with the ARRIVE guidelines. All experiments were performed on C57BL/6J mice.

Timed-pregnant dams were sacrificed by cervical dislocation on E14.5 (with day of plug defined as E0.5), embryos were removed and decapitated in HBSS on ice. Sections of anterior cortices corresponding to the primary motor area were dissected and pooled within litters. Cells were washed with HBSS and dissociated with 50 μL 0.5% Trypsin, 0.25% EDTA at 37 °C for 15 minutes. Dissociation was ceased by addition of 100 μL FBS and cells were pelleted and resuspended in Neurobasal Media (no L-glutamine, no Phenol red) supplemented with 2% B27, 0.5 mM Glutamax and 0.1% Penicillin/Streptomycin. Cells were cultured in 24 well plates containing 13 mm round coverslips coated with sterile filtered 50 μg/mL Poly-D-Lysine at a density of 10^−5^ per well, and maintained at 37 °C, 5% CO_2_, 60% humidity. For Netrin-1 assays, recombinant mouse Netrin-1 (R&D systems), or PBS control, was diluted in plating media and added drop-wise to wells at a final concentration of 200 ng/mL. Reconstituted Netrin-1 was stored at −80 °C and used within 3 months of reconstitution.

### RNAi

All siRNA transfections were performed using RNAiMAX (Invitrogen) according to the manufacturer’s instructions. Knockdown of *Rad51* was performed using a combination of two siRNAs (10 nm each per well); coding region *Rad51*; 5′-GGUAAAUCACCAACCAGGUAGU-3′, 5′ UTR *Rad51*; 5′-GUGGCCAGGAAGACUGAAAUU-3′, Scramble control; 5′-GGCAGACUACGUUACUACAGA-3′. We initially screened three different siRNAs both individually and in combination, targeted to either the 5′ UTR or coding region of *Rad51*, validated their ability to reduce the levels of *Rad51* mRNA and protein, and based on this, selected a duplex which gave the most significant reduction of *Rad51* expression (see Supplementary Fig. S2). At 4 hours after plating, E14.5 cortical neurons were treated with either siRNA specific for *Rad51* (*Rad51*siRNA), a scrambled control siRNA (scramsiRNA), or transfection reagent alone. Netrin-1 assays were performed 2 days *in vitro* (DIV) post-siRNA, which was determined to be sufficient time for significant siRNA-mediated reduction of *Rad51* mRNA and protein (see Supplementary Fig. S2).

### Rad51 Overexpression Assays

The *RAD51* open reading frame was PCR-amplified from human cDNA and cloned into a plasmid vector allowing the expression of an EYFP-RAD51 fusion transcript in mammalian cells (see Supplementary data for details). Sequence integrity and orientation was confirmed before large scale plasmid DNA amplification. Plasmid DNA was extracted by alkaline lysis, and DNA was eluted in 10 mM TE pH 8.0. Neurons were transfected at 1 DIV with either EYFP-RAD51, or EYFP-R250Q DNA constructs, or EYFP controls (500 ng/well), or vehicle alone, using Lipofectamine 2000 diluted in plating media. After 24 hours cultures were assayed for EYFP fluorescence.

### RNA Extraction and Quantitative PCR (qPCR)

Total RNA was extracted using Trizol (Gibco) and Direct-zol RNA MiniPrep kit (Zymo), followed by Dnase-treatment using Ambion DNA-free DNase. RNA purity and quality was analysed using a NanoDrop^®^ ND‐1000 spectrophotometer, and quantitated on a Qubit^®^ 2.0 Fluorometer using Qubit^®^ RNA Assay Kit. RNA was reverse transcribed in 25 μl reactions using Superscript III reverse transcriptase as per manufacturers instructions. Resulting cDNA was stored at ‐80 °C until required. All primers for qPCR analysis were designed using PrimerBLAST (http://www.ncbi.nlm.nih.gov/tools/primerblast), sequences are listed in supplementary methods. All qPCR reactions were performed in triplicate in 10 μL volume containing 2X SYBR Master mix (Bioline) and 1 μL of cDNA template under standard thermal cycling conditions on a Viia7 Real Time PCR System (Applied Biosystems). No template control and no RT control reactions were included in each qPCR run, and gene expression was normalised to two reference genes: TATA box binding protein (*Tbp*) and phosphoglycerate kinase 1 (*Pgk1*). Fold change was determined in comparison to EYFP transfected controls using the 2−ΔΔCT relative quantification method^36^.

### Immunocytochemistry

Immunocytochemistry was performed using standard protocols. Briefly, cells were fixed in 4% Paraformaldehyde in PBS, permeabilised with 0.25% Triton-X-PBS, blocked in 2% serum, 2% BSA, 0.1% Triton-X- PBS for 30 min, then incubated in primary antibody overnight at 4 °C. For detection of primary antibodies, cells were incubated with species-specific Alexa Fluor 488- or 594-conjugated secondary antibodies (1:500) for 90 min, and nuclei were counterstained with DAPI. Antibodies used were: rabbit anti-Rad51, 1:1000 (Abcam, ab63801), rat anti-β-III tubulin (TuJ1, monoclonal; R&D Systems) chicken anti-TuJ1 (Abcam, ab107216).

### Data analysis/Statistics/Imaging/NeuronJ

Neuron tracings were performed using the NeuronJ plugin^37^ for ImageJ software (v1.46r, National Institutes of Health). Neurites were deemed to be the primary axon if they were at least twice the length of the cell body diameter, and the longest process. For secondary neurite measurements, data were separated into two categories: ≥5 μm = neurites, <5 μm = filopodia. Comparisons between groups was performed using One-way ANOVA followed by Tukey’s multiple comparison test. Confocal images were acquired on a Zeiss LSM710 microscope with a 63X objective, acquiring optical sections of 1 μm.

## Additional Information

**How to cite this article:** Glendining, K. A. *et al*. A novel role for the DNA repair gene *Rad51* in Netrin-1 signalling. *Sci. Rep.* **7**, 39823; doi: 10.1038/srep39823 (2017).

**Publisher's note:** Springer Nature remains neutral with regard to jurisdictional claims in published maps and institutional affiliations.

## Supplementary Material

Supplementary Information
