# Supplementary material for: A novel role for the DNA repair gene Rad51 in Netrin-1 signalling
Source: Sci Rep. 2017 Jan 6;7:39823. doi: 10.1038/srep39823 (PMC5216413; doi:10.1038/srep39823)
Supplement: Supplementary Information [file srep39823-s1.pdf]

Supplementary information to accompany:

A novel role for the DNA repair gene *Rad51* in Netrin-1 signalling

Glendining KA[1], Markie D[2], Gardner RJM[4], Franz EA[3], Robertson SP[4], Jasoni CL[1]

Supplementary Methods:

EEYFP-RAD51 fusion construct

A Gateway Destination vector allowing expression of EEYFP fusion constructs in mammalian cell lines was made by first replacing the multiple cloning site (from the HindIII to ApaI sites) of pcDNA4/TO/*myc*-HisA (Invitrogen) with a PCR fragment generated from Gateway cassette A (Invitrogen). A PCR fragment for the EEYFP gene, including an optimal Kozak sequence, was then inserted at the HindIII site upstream of the Gateway cassette to produce the plasmid pDEST/TO/EEYFP/myc-His. Open reading frames inserted in the correct reading frame into the Gateway cloning site of this plasmid vector will be expressed under the control of the CMV promoter as fusion proteins with the EEYFP sequence upstream, and the myc and 6xHis epitopes downstream. In cell lines engineered to express the tet repressor, expression of the fusion protein should be tetracycline inducible owing to the presence of the TetO2 sequence between the promoter and the transcriptional start site, otherwise expression will be constitutive.

The full length *RAD51* open reading reading frame (including it's native termination codon to avoid inclusion of the myc and 6xHis epitopes in the expressed protein) was PCR amplified from human cDNA (QUICK-Clone™ II Human Universal cDNA, ClonTech) using primers containing the attB1 and attB2 tags. It was cloned using the Gateway BP reaction (Invitrogen) into the pDONR201 plasmid (Invitrogen) and a sequence verified clone chosen. The *RAD51* sequence was then transferred to the pDEST/TO/EEYFP/myc-His plasmid using the Gateway LR reaction to produce pEEYFP/RAD51.

The mutant *RAD51* R250Q gene was produced by overlap PCR. In brief, the *RAD51* insert from the sequenced-verified pDONR201-RAD51 plasmid was initially amplified as two overlapping fragments in separate PCR reactions. The 24-base overlapping segment was specified by complementary primers for each reaction, which each contained an appropriate single base substitution to specify the R250Q mutation. The previous *RAD51* primers containing the attB1 and attB2 sites were used as the second primer in respective reactions. After removal of the original primers, the two fragments were mixed, and a second round of PCR undertaken with just the two external primers. The resulting fragment was cloned using the Gateway BP reaction into the pDONR201 plasmid and a sequence verified clone that contained only the R250Q change was chosen. The *RAD51* R250Q sequence was then transferred to the pDEST/TO/EYFP/myc-His plasmid using the Gateway LR reaction to produce pEYFP/RAD51-R250Q

Cell staging analysis

Neuronal differentiation of cultured cells was assessed following 24 hr treatment with either Rad51 siRNA, scramble control siRNA, or Vehicle alone, and then an additional 24 hour incubation with Netrin1, or PBS. For morphological analysis, cells were fixed in 4% paraformaldehyde in PBS, pH 7.4, and immunostained for neuron-specific βIII-tubulin (1:1000, chicken anti-mouse; Abcam), and differentiation was classified by three stages of cell morphology: *Stage 1*, lamellipodia/filopodial protrusion, but no neurites; *Stage 2*, neurites present but extending ≤ 10 μm from the cell body ; *Stage 3*, axon specification as defined by the longest process extending ≥10 μm from the cell body. Stages of differentiation were analysed from >450

neurons per condition, with data averaged across triplicate wells, from 4 individual cultures.

Western Blot

Cultured neurons were lysed on ice in RIPA buffer (Pierce, ThermoFisher), containing Protease inhibitors (Complete, EDTA free;Roche) and Phosphatase inhibitors (250nm Sodium Fluoride, Tetra Sodium Pyrophosphate, 50mM Na_2Glycerophosphate and 5mM activated Sodium Vanadate). Lysates were pelleted at 14,000rpm, 4 °C, protein concentration was determined by DC protein assay (Biorad), and supernatants were stored at -80 °C. For Western blot, protein was denatured in Laemmli buffer at 95 °C for 5 min and subjected to SDS-PAGE analysis. Separated proteins (10ug/lane) were transferred to PVDF membrane, blots were blocked for 30 min using Odyssey Blocking Buffer (LI-COR Bioscience) and then incubated with primary antibody overnight at 4 °C. Antibodies used were rabbit anti-beta tubulin (Abcam, 1:3,000), Anti-RAD51 mouse monoclonal (ThermoFisher, 1:500), IRDye infrared secondary antibodies Goat anti-Rabbit IgG 650, and Goat anti-Mouse IgG 800 (LI-COR Biosciences) were used at concentration 1:3,000. Blots were analysed using the Odyssey Infrared imaging system (LI-COR Biosciences) and ImageStudio software.

qPCR Primer sequences

Pgk1_Fwd:CTCCGCTTTCATGTAGAGGAAG Pgk1_Rev: GACATCTCCTAGTTTGGACAGTG, Tbp_Fwd: GAAGAACAATCCAGACTAGCAGCA, Tbp_Rev:

CCTTATAGGGAACTTCACATCACAG, Unc5a_Fwd;

CTCCTGGGCATAGTCCTCACT, Unc5a_Rev; CAGCACGGGCTTGTTCTTG,

Unc5b_Fwd; CTGGAGGACACACCTGTAGCACTG, Unc5b_Rev;

GAGGCGTAGGTTGTGGTAACTGTC, Unc5c_Fwd;

AGGCAGTGCAGGGACAAT,

Unc5c_reverse; CCAGGGCATAGGTACTGAGG, Unc5d_ Fwd;

AGACCTCCATAGATGTGTGGA, Unc5d_ Rev;

AGCGGAAGTGACCCATGACGT,

Dcc_ Fwd; GCTTTTGTCTCAGCCAGGACCCAC, Dcc_R;

ACAGACACGGGAAGCAAAGGGG,

Rad51_ Fwd; CCAGACCCAGCTCCTTTACC, Rad51_ Rev;

CACTGCGACACCAAACTCATC

Supplementary Figure Legends:

Fig. S1. Rad51 mRNA and protein expression in neurons was not altered in response to Netrin1.

(A) Bar graph showing qPCR analysis of Rad51 mRNA expression levels in cultured cortical neurons after treatment with Netrin-1 (Net1) for 2 or 24 hours in comparison to PBS treated controls. Values are mean ± SEM (B) Representative Western blot analysis of 37 kDa Rad51 protein levels after 24 hours treatment with either PBS or Netrin-1 (Net1). Beta-tubulin (β-tub, 55 kDa) shown as loading control. (C) Bar graph depicting quantification of the Western blot in (B). Arbitrary fluorescence signal after normalisation to loading control beta-tubulin. Values shown are mean ± SEM.

Fig. S2. Neuronal differentiation and axon elongation was not altered by knockdown of Rad51.

Bar graph showing stage of maturation of cultured neurons (as determined by morphological criteria, see Methods) treated with either an siRNA targeting Rad51 (*Rad51* siRNA), scramble control siRNA (Scram siRNA), or vehicle, followed by addition of Netrin-1 (Net) or PBS. Values shown are means. Number of cells analysed are indicated at base of bars.

Fig. S3. Validation of siRNA-mediated knockdown of *Rad51* expression in cultured neurons.

(A) Bar graph showing qPCR quantification of *Rad51* mRNA following treatment with different siRNAs targeting the 5' UTR or coding region of *Rad51*. (B) Representative Western blot of 37 kDa Rad51 protein after 48 hours siRNA treatment. (C-D) Representative images of cultured neurons treated with either Scram siRNA control (C) or *Rad51* siRNA5 (D). Rad51 immunofluorescence (green), DAPI (blue).

Fig. S4. TuJ1/YFP-RAD51 co-expression

Representative images of TuJ1-immunostained axons (red) treated with PBS (A-C) or Netrin-1 (D-F) following transfection with EYFP (green) fused to an empty plasmid (Empty, A, D), wildtype RAD51 (wtRAD51, B, E), or the RAD51 R250Q mutant (R250Q, C,F). Scale bar is 5μm.

Fig. S1

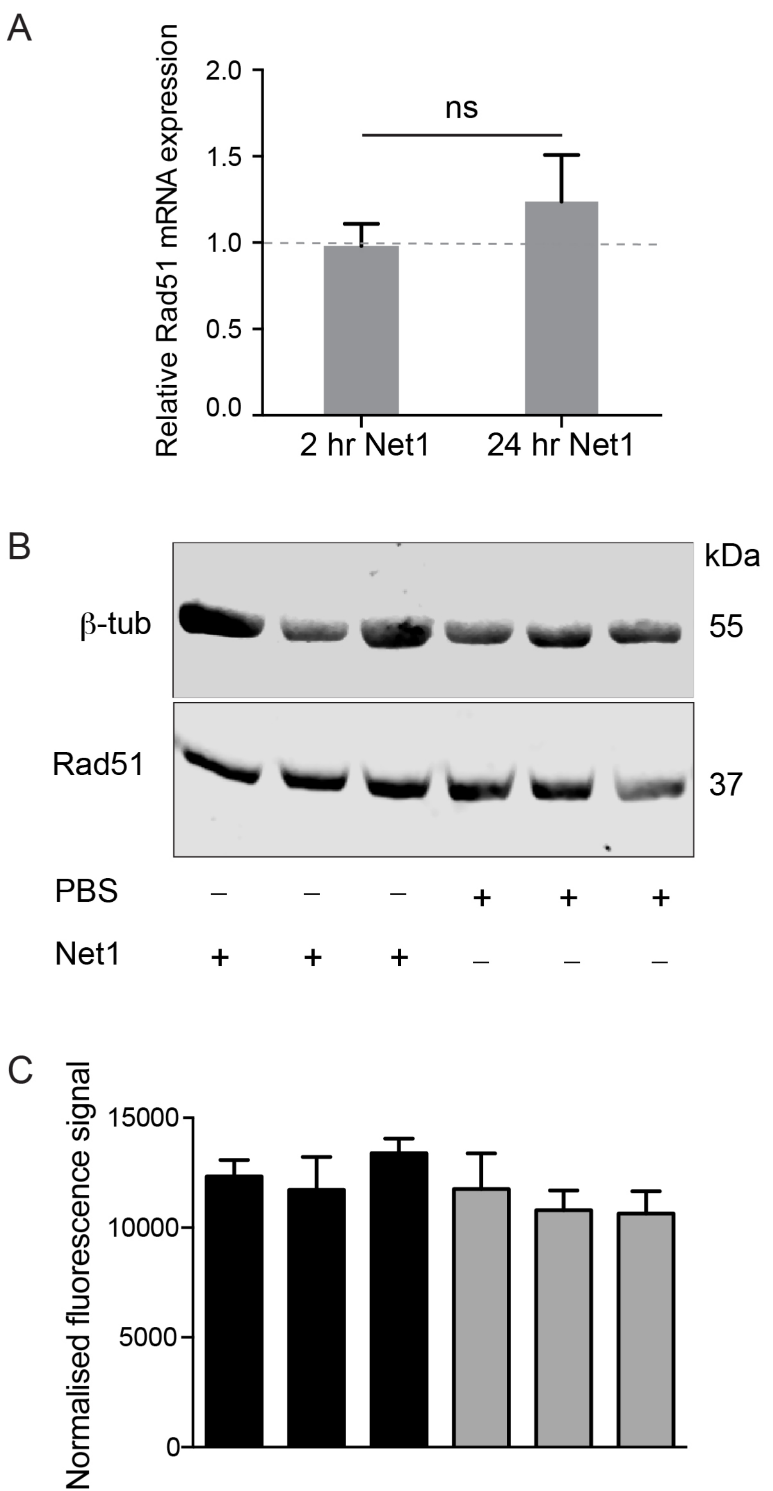
A
Relative Rad51 mRNA expression
2.0
1.5
1.0
0.5
0.0
ns
2 hr Net1
24 hr Net1
B
kDa
β-tub
55
Rad51
37
PBS – – – + + +
Net1 + + + – – –
C
Normalised fluorescence signal
15000
10000
5000
0

Fig. S2

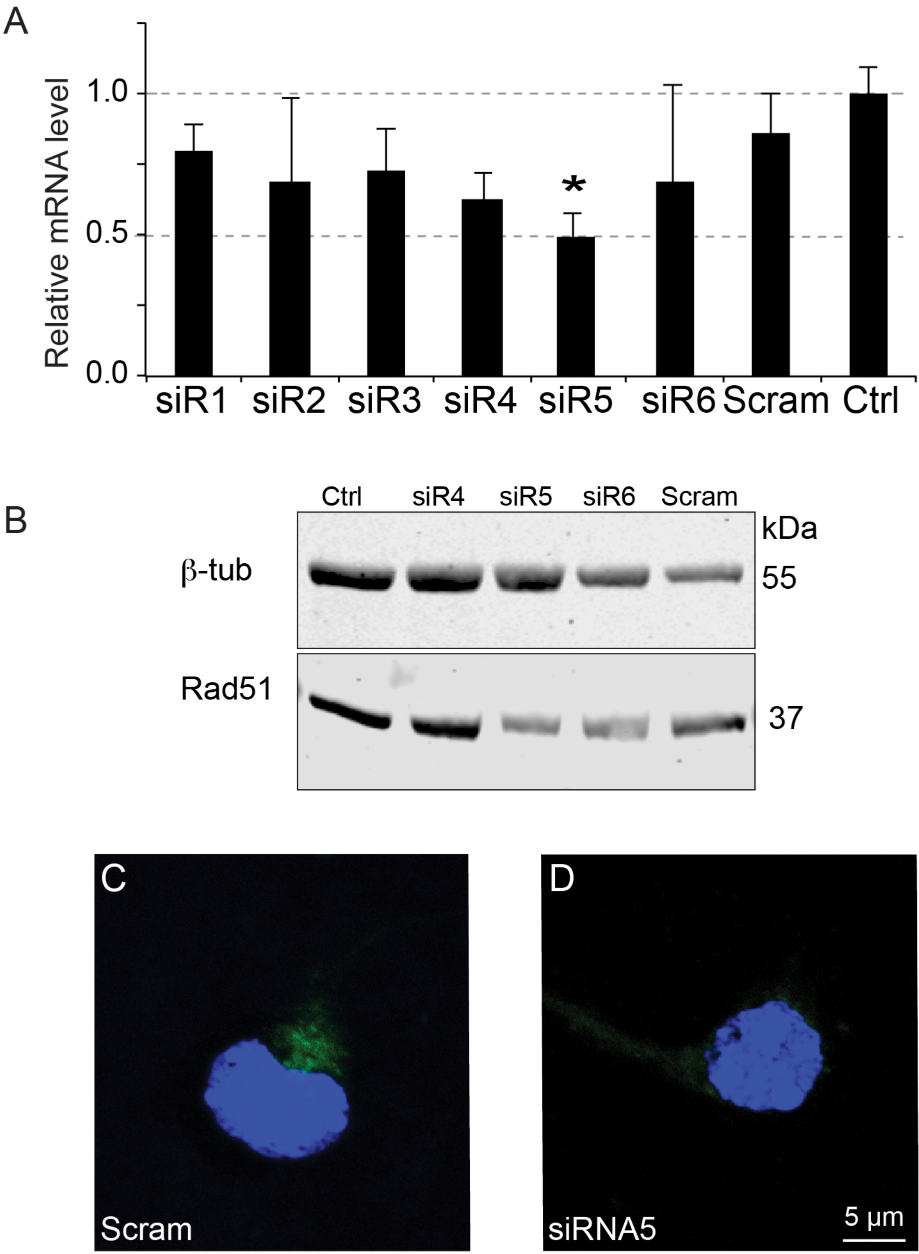
A
Relative mRNA level
1.0
0.5
0.0
*
siR1
siR2
siR3
siR4
siR5
siR6
Scram
Ctrl
B
Ctrl
siR4
siR5
siR6
Scram
kDa
β-tub
55
Rad51
37
C
D
Scram
siRNA5
5 µm

Fig. S3

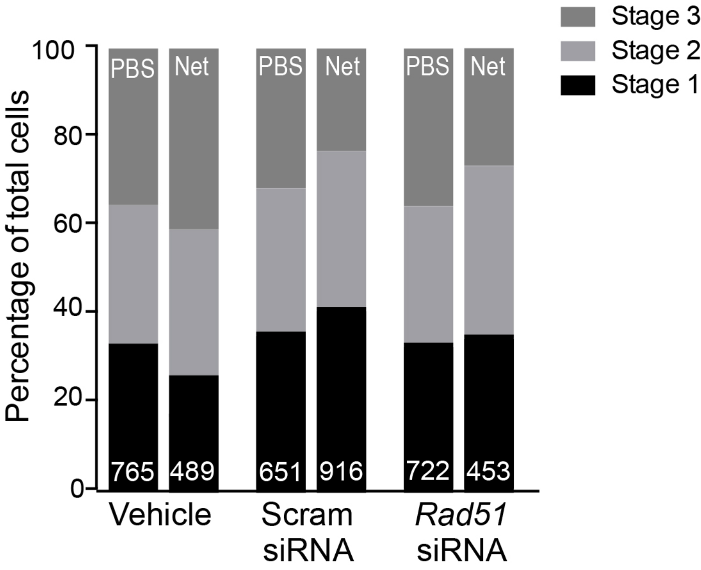

Stage 3
Stage 2
Stage 1
100
80
60
40
20
0
Percentage of total cells
PBS
Net
PBS
Net
PBS
Net
765
489
651
916
722
453
Vehicle
Scram siRNA
Rad51 siRNA

Fig. S4

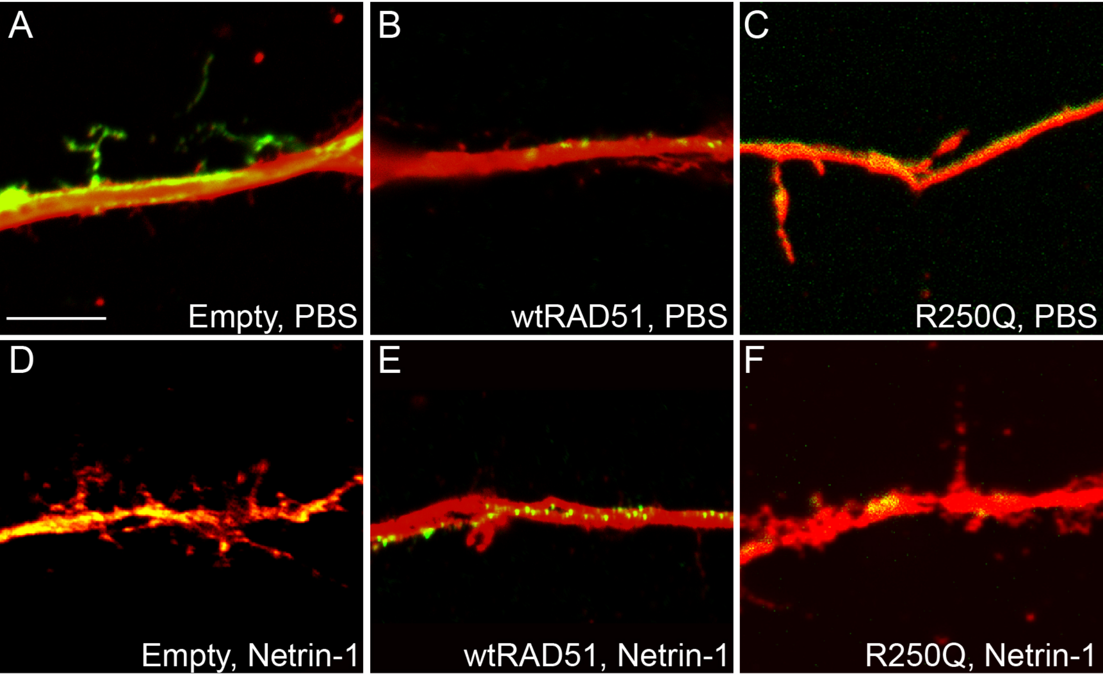
A
Empty, PBS
B
wtRAD51, PBS
C
R250Q, PBS
D
Empty, Netrin-1
E
wtRAD51, Netrin-1
F
R250Q, Netrin-1
